# Pediatric vaccination in pharmacies is not associated with delayed well-child visits among commercially insured children

**DOI:** 10.1093/haschl/qxaf028

**Published:** 2025-02-10

**Authors:** Shiven Bhardwaj, Nina Galanter, Lucas A Berenbrok, Parth D Shah, Jennifer L Bacci

**Affiliations:** The Comparative Health Outcomes, Policy, and Economics (CHOICE) Institute, School of Pharmacy, University of Washington, Seattle, WA 98195, United States; Department of Biostatistics, School of Public Health, University of Washington, Seattle, WA 98195, United States; Department of Pharmacy and Therapeutics, School of Pharmacy, University of Pittsburgh, Pittsburgh, PA 15261, United States; The Comparative Health Outcomes, Policy, and Economics (CHOICE) Institute, School of Pharmacy, University of Washington, Seattle, WA 98195, United States; Hutchinson Institute for Cancer Outcomes Research (HICOR), Fred Hutchinson Cancer Center, Seattle, WA 98109, United States; The Comparative Health Outcomes, Policy, and Economics (CHOICE) Institute, School of Pharmacy, University of Washington, Seattle, WA 98195, United States

**Keywords:** pharmacy-based vaccination, pediatric immunization, pharmacy-administered vaccination, pharmacy-administered pediatric vaccination, Vaccines for Children program

## Abstract

Pediatric vaccination rates in the United States lag national goals. Policies that expand pharmacy-based vaccinations among children could help improve vaccination rates. Opponents argue, however, that such policies will result in delayed or missed well-child visits as most children receive routine vaccinations in primary care settings. We evaluated the likelihood of having a timely well-child visit following a routine vaccination in pharmacies and primary care settings among children aged 4–17 years. We conducted a retrospective cohort analysis with commercial claims data from 2016–2019, using conditional logistic regression models. A timely well-child visit was defined as one within 12 months after a preceding well-child visit for primary analysis and 15 months for secondary analysis. Approximately 95% of the sample consisted of children with influenza among their index vaccine(s). The odds of having a timely well-child visit were similar between children who received vaccines in pharmacies and those who received them in primary care settings. Findings suggest that guardians or parents who choose pharmacy-based pediatric vaccinations for their commercially insured children do not forgo well-child visits and may actually be more likely to obtain a timely well-child visit. Extending pharmacy-based vaccinations to patients of all ages can help improve pediatric vaccination rates.

## Introduction

Pediatric and adolescent vaccination rates remain below national target coverage levels in the United States. The country failed to meet several Healthy People 2020 targets related to childhood vaccines and there has been little to no detectable change for several Healthy People 2030 targets to date.^1,2^ Suboptimal vaccination rates have the potential to adversely affect herd immunity and may be contributing to recent outbreaks of vaccine-preventable diseases like measles.^3^

Randomized trials and observational studies suggest that pharmacy-based vaccination in adults result in an increased uptake of routine vaccinations recommended by the Advisory Committee on Immunization Practices (ACIP).^4-7^ As such, expanding pharmacy-based vaccination among children could also effectively complement existing pediatric vaccination efforts.

Pharmacy practice, including vaccine administration, is regulated by boards of pharmacy or equivalent regulatory bodies in each US state and jurisdiction. Although all US states and jurisdictions allow pharmacies to administer adult vaccinations, many significantly restrict the scope of pharmacy practice on pediatric vaccinations with respect to (1) pharmacists’ authority to prescribe vaccines and pharmacies’ authority to administer them; (2) the type of vaccines pharmacies are authorized to administer; and (3) which patients, based on their age, pharmacies are authorized to vaccinate. In 2024, 39 states had at least 1 age-based restriction on pharmacy-based vaccination and 29 states allowed pharmacists to administer influenza vaccine to everyone above the age of 3 years.^8^ Children who reside in states with permissive age-based pharmacy vaccination policies are more likely to receive routine vaccinations in pharmacies.^9^

Expansion of pharmacy-based pediatric vaccination faces significant opposition from medical organizations, which argue that such expansion will lead parents/guardians to delay or miss well-child visits for their children, fracturing primary care.^10^ Well-child visits are regularly scheduled preventive care visits with primary care providers, which include routine vaccination, among other services.^11^ While assertions of delayed or missed well-child visits warrant careful consideration, there is no rigorous evidence to support or refute this claim. Among adults, an increase in pharmacy-based vaccinations has not been associated with decreased primary care use, suggesting that adult vaccination rates increased without a reduction in other preventive services.^12^ While patterns of primary care use vary significantly between pediatric and adult populations, evidence from adult population might extend to children, and justifies further investigation.

We aimed to evaluate whether pharmacy-based pediatric vaccination was associated with delayed or missed well-child visits among a cohort of children aged 4–17 years. We hypothesized that children who receive a routine vaccination in a pharmacy are not less likely to have a timely well-child visit compared with children who receive a routine vaccination in a primary care setting.

## Data and methods

### Data source

We conducted a retrospective cohort analysis using Merative Marketscan medical and pharmacy claims data. Marketscan is a health insurance claims database for commercially insured individuals and their dependents throughout the United States, representing 25 to 27 million persons each year under the age of 65 years.^13^ It consists of a wide range of large private employers and health plans. The database captures outpatient, inpatient, facility, and pharmacy claims for enrollees, and provides deidentified demographic data, including state of residence, sex, and age. It does not include information on individuals’ race, ethnicity and location by county or zip code.

Pharmacies are prohibited from, or have significant challenges in, billing state Medicaid agencies for vaccinations that are available through the Vaccines for Children (VFC) program. As such, most pharmacies do not participate in the VFC program or provide vaccinations to children who are uninsured or are insured by Medicaid and the Children's Health Insurance Program (CHIP)^14^; thus, exclusion of Medicaid claims from our study would not introduce bias.

### Study sample

We divided our study population into 2 age groups, 4–8 years and 9–17 years, based on the ACIP vaccine recommendations. Using ACIP's 2017 and 2018 immunization schedules,^15,16^ we identified target vaccines routinely given for each age group, as follows: (1) influenza, varicella, MMR (measles, mumps, and rubella), IPV (inactivated poliovirus), and DTaP (diphtheria, tetanus, and acellular pertussis) vaccines for ages 4–8 years and (2) influenza, Tdap (tetanus, diphtheria, and acellular pertussis), meningococcal, and HPV (human papillomavirus) vaccines for ages 9–17 years. Details on claim identification for target vaccines and well-child visits are available in Tables A1–A3 in the supplemental materials.

Within the 2 age groups, we compared 2 cohorts—pharmacy and office-based primary care setting. The pharmacy cohort consisted of children who had a pharmacy claim for a target vaccine from January 1, 2017, through December 31, 2018. The primary care cohort consisted of individuals who had an outpatient claim for a target vaccine in a primary care setting within the same period and did not have any pharmacy claims for vaccines during the study period or within 1 year of the target vaccine claim. A list of primary care outpatient settings included in primary care cohort can be found in Table A4. The service date of the target vaccine claim served as the index date. If there was more than 1 claim for a target vaccine within the study period, the earliest service date was used as the index date. The study period was limited to before the COVID-19 pandemic to minimize the risk of reverse causality due to 2 potential confounders. First, parents who could not schedule a well-child visit during the pandemic may have sought vaccinations at pharmacies. Second, the Public Readiness and Emergency Preparedness (PREP) Act's third amendment became effective on August 24, 2020, authorizing trained pharmacy professionals in all US jurisdictions to independently prescribe and administer any ACIP-recommended vaccine to patients as young as 3 years old.^17^

Both the primary care and pharmacy cohorts excluded individuals who (1) did not have a well-child visit within 12 months before their index date (ie, qualifying well-child visit), (2) had any inpatient stays within 3 months of their index date, or (3) did not have continuous insurance enrollment of 12 months prior to the index date and 15 months after the index date. A 12-month continuous enrollment was necessary to reliably identify a qualifying well-child visit, whereas the 15-month continuous enrollment after the index date was necessary to identify the outcome. We allowed qualifying well-child visits to be on the same day as the index date as this includes many real-world scenarios where patients may receive a routine vaccine during their well-child visit. The qualifying well-child visit served 2 purposes: (1) ascertaining whether a subsequent well-child visit was delayed and (2) establishing access to primary care services for the study population. All inclusion criteria with justifications are listed in Table A5.

**Figure 1.**
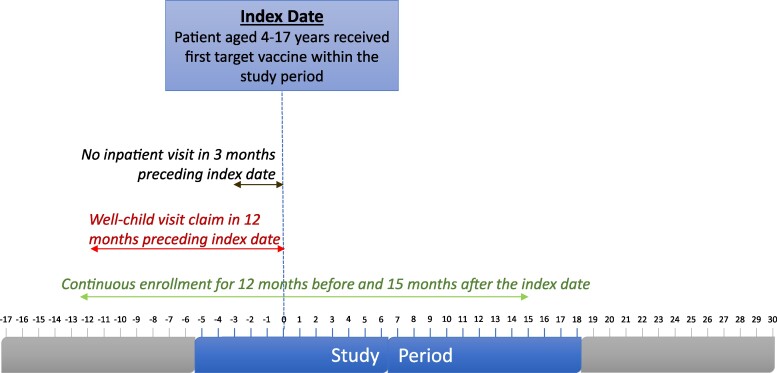
Patient selection criteria relative to study period.

**Figure 2.**
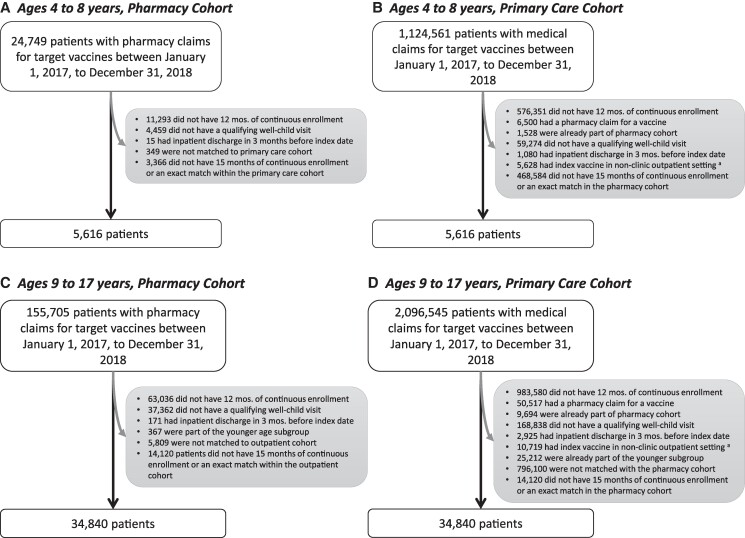
Flow diagram of cohort construction sample based on selection criteria. A. Pharmacy cohort, ages 4 to 8 years, B. Primary care cohort, ages 4 to 8 years, C. Pharmacy cohort, ages 9 to 17 years, D. Primary care cohort, ages 9 to 17 years. ^a^Non-clinic outpatient settings included pharmacy, school, patient home, mobile unit, walk-in retail health clinic, urgent care facility, inpatient hospital, emergency room, ambulatory surgical center, birthing center, military treatment facility, skilled nursing facility, community mental health center, residential substance abuse facility, psychiatric residential treatment center, mass immunization center, comprehensive outpatient rehab facility, independent laboratory and other/unknown.

Figure 1 depicts an example of an eligible patient and application of selection criteria and Figure 2 shows numbers of individuals excluded due to each selection criterion in both cohorts.

### Measures

#### Primary outcome: timely well-child visit within 12 months

The primary outcome was whether a well-child visit claim was recorded within 12 months of the preceding qualifying well-child visit between pharmacy and primary care cohorts. We selected a 12-month period based on the recommended frequency for annual well-child visits by the American Academy of Pediatrics.^18^ We compared the odds of having a timely well-child visit in the pharmacy group with the odds of having a timely well-child visit in the primary care group. This outcome was assessed separately for the 2 age groups (4–8 years and 9–17 years).

#### Secondary outcome: timely well-child visit within 15 months

The secondary outcome was whether a well-child visit claim was recorded within 15 months of the preceding qualifying well-child visit between pharmacy and primary care cohorts. We assessed this extended period to accommodate delays in scheduling or attending subsequent well-child visits. We could not explore an outcome of lengthier time between qualifying and subsequent well-child visits as such analysis would have included earlier months of the COVID-19 Public Health Emergency (PHE). For the secondary outcome, we used individuals from the matched sample used in primary outcome but limited our analysis to pairs who both had 15 months of continuous enrollment after their index date. To ensure that the subgroup in secondary analysis was not healthier than the one in primary analysis due to longer continuous enrollment, we used the 15-month continuous enrollment criterion for both primary and secondary analyses.

### Covariates

We identified covariates for all analyses a priori based on available data and literature review.^9,19-22^ The primary care cohort was exactly matched with the pharmacy cohort on the following: (1) sex, (2) age strata (4–6 years and 7–8 years for the younger group, 9–12 years and 13–17 years for the older group), (3) rurality, (4) US state or jurisdiction of residence, (5) type of health insurance plan (Preferred Provider Organization, Consumer-Directed Health Plan, High-Deductible Health Plan, Health Maintenance Organization, Point of Service, Comprehensive, Exclusive Provider Organization, or Point of Service with Capitation), (6) Pediatric Comorbidity Index, (7) influenza vs non-influenza index vaccine, and (8) year of the index date. We determined rurality based on Metropolitan Statistical Area (MSA), where a value of zero denoted rurality. The MSA represents a geographic region with at least 1 urban area with population of 50 000 or greater.^23^ Areas without an MSA designation (ie, MSA = 0) are largely rural. We calculated a Pediatric Comorbidity Index (PCI) score for everyone included in our analysis to account for comorbidity burden and its association with hospitalization based on International Classification of Diseases, Tenth Revision (ICD-10), values recorded in inpatient and outpatient primary care claims during 1 year before the index date.^24^ We matched on the index date year to adjust for the imbalance in distribution of index date year between the 2 cohorts (Table A6). More details on covariate selection and use for matching are described within Section 2 of the supplemental materials.

### Analysis

#### Primary and secondary outcomes analyses

All analyses compared the odds of receiving a timely well-child visit between primary care and pharmacy cohorts. We used conditional logistic regression with the binary outcome of having a timely well-child visit. Regression was conditioned on matched pair identified through exact matching.

#### Exploratory subgroup analyses

We conducted several subgroup analyses for both primary and secondary outcomes. Within the primary analysis cohort (well-child visit within 12 months of the qualifying visit) we compared pharmacy and primary care groups among those who received an influenza vaccine on their index date (subgroup analysis 1) and those who did not have an influenza vaccine on their index date (subgroup analysis 2). Similarly, for the secondary analysis cohort (well-child visit within 15 months of the qualifying visit), we compared the pharmacy and primary care groups among those who received an influenza vaccine on their index date (subgroup analysis 3) and those who did not have an influenza vaccine on their index date (subgroup analysis 4). We conducted these subgroup analyses to explore whether results varied by use of influenza vaccine, as its seasonal schedule may have motivated some individuals with well-child visits during summer months to receive the vaccine at a pharmacy. Table A7 details all analyses conducted.

All data cleaning and analyses were conducted in SAS version 9.4 (SAS Institute, Cary, NC) and R version 4.3.2 (R Foundation for Statistical Computing, Vienna, Austria). All test statistics were 2-sided and used a critical alpha = .05. We used the Strengthening the Reporting of Observational Studies in Epidemiology (STROBE) reporting guidelines for this study.

### Ethics and human subjects review

The Institutional Review Board of the University of Washington exempted this study from human subjects review.

## Results

### Analytic sample characteristics

#### Exact matching

The matched cohorts consisted of 11 232 individuals in the group aged 4 to 8 years (5611 individuals in each cohort) and 69 680 individuals in the group aged 9 to 17 years (34 840 individuals in each cohort). Both age groups had a similar distribution of sex, rurality, health plan type, US state of residence, and mean and median time between qualifying and follow-up well-child visits (Table 1). The mean and range of the PCI scores did not differ between each age group's pharmacy and primary care cohorts but differed between the 2 age groups.

**Table 1.** Participant characteristics in primary and secondary analyses cohorts.

	Ages 4–8 years (*n* = 11 280)		Ages 9–17 years (*n* = 69 680)	
	Pharmacy cohort (*n* = 5616)	Primary care cohort (*n* = 5616)	Pharmacy cohort (*n* = 34 840)	Primary care cohort (*n* = 34 840)
Mean (SD) age,^a^ y	6.53 (1.35)	6.36 (1.52)	13.52 (2.46)	13.00 (2.64)
4–6 y	2340 (41.67%)		N/A	
7–8 y	3276 (58.33%)		N/A	
9–12 y	N/A		12 580 (36.11%)	
13–17 y	N/A		22 260 (63.89%)	
Sex, female, *n* (%)	2695 (47.99%)		17 494 (50.21%)	
Rural Metropolitan Statistical Area, *n* (%)	405 (8.25%)		2589 (8.50%)	
Health plan type, *n* (%)				
PPO	2808 (51.40%)		17 458 (51.09%)	
CDHP	1035 (18.95%)		6539 (19.13%)	
HDHP	891 (16.31%)		5113 (14.96%)	
HMO	524 (9.59%)		3590 (10.50%)	
POS	83 (1.52%)		773 (2.26%)	
Comprehensive	65 (1.19%)		416 (1.22%)	
EPO	40 (0.73%)		218 (0.64%)	
POS with capitation	17 (0.31%)		67 (0.20%)	
Region,^a,b^ *n* (%)				
Northeast	464 (8.26%)		3795 (10.89%)	
North Central	1314 (23.40%)		8541 (24.52%)	
South	2325 (41.40%)		15 694 (45.05%)	
West	1505 (26.8)		6620 (19.00%)	
Unknown	8 (0.14%)		186 (0.53%)	
Mean (SD) Pediatric Comorbidity Index^c^	0.53 (1.05)		1.0 (1.9)	
Mean (SD) time between well-child visits,^a^ d	342.7 (37.0)	333.4 (47.58)	332.5 (48)	322.60 (61.41)
Median (IQR) time between well-child visit and index date,^a^ d	356.00 (330–382)	351.00 (312–390)	350.00 (312–388)	348 (293.00–403.00)
Index year, *n* (%)				
2017	2104 (37.46%)		16 140 (46.33%)	
2018	3512 (62.54%)		18 700 (53.67%)	

^a^Not a covariate.

^b^States were used to match the cohort. Table A9 consists of cohort distribution by states.

^c^Pediatric comorbidity index scores ranged from 0 to 15 for age group 4 to 8 years, and 0 to 26 for age group 9 to 17 years.

Abbreviations: CDHP, Consumer-Directed Health Plan; EPO, Exclusive Provider Organization; HDHP, High-Deductible Health Plan; HMO, Health Maintenance Organization; N/A, Not Applicable; POS, Point-of-Service; PPO, Preferred Provider Organization.

#### Vaccination claims

Within the pharmacy cohort for each age group, influenza vaccination claims accounted for over 95% of the sample on their index date (Table A8). The MMR, varicella, and DTaP vaccines accounted for less than 0.50% of patients in the younger age group, and Tdap, meningococcal, and HPV vaccines comprised 1.02%, 2.24%, and 1.37% of the sample in older age group, respectively. Among the primary care cohort for both age groups, influenza vaccine was most common among index vaccines (99.79% in ages 4 to 8 years and 96.44% in ages 9 to 17 years). In the younger age group, greater than 5% of the sample received DTaP (15.06%), MMR (5.20%), varicella (5.40%), and combinational MMR and varicella (9.73%), whereas only 2.66% of the sample received the IPV vaccine. Among older children in the primary care group, meningococcal vaccine (13.11%) was most common after influenza, followed by Tdap (5.34%) and HPV (2.42%) vaccines.

### Primary analysis: timely well-child visit within 12 months of qualifying well-child visit

Results are shown in Table 2. For the younger age group, children who received a routine vaccination at a pharmacy had a similar likelihood of having a timely well-child visit compared with children who received a routine vaccination in a primary care setting (odds ratio [OR] = 0.94; 95% CI: 0.86–1.04), whereas in the older age group, the likelihood of a timely well-child visit was slightly higher in the pharmacy cohort (OR = 1.04; 95% CI: 1.00–1.08). Among those who received an influenza vaccine on their index date (subgroup analysis 1), the likelihood of a timely well-child visit was similar between the pharmacy and primary care cohorts in the younger age group (OR = 0.94; 95% CI: 0.87–1.03), whereas the likelihood of a timely well-child visit was slightly higher in the pharmacy cohort among the older age group (OR = 1.04; 95% CI: 1.00–1.01). In individuals who did not receive an influenza vaccine on their index date (subgroup analysis 2), the likelihood of a timely well-child visit was similar in the pharmacy and primary care cohorts (OR = 1.01; 95% CI: 0.83–1.22).

**Table 2.** Timely well-child visits in pharmacy and primary care cohorts.

Group	*n*/Total *n*	Odds ratio	95% CI
Primary analysis			
Ages 4 to 8 years			
Primary care cohort	1126/5616	Reference	Reference
Pharmacy cohort	1074/5616	0.94	0.86–1.04
Ages 9 to 17 years			
Primary care cohort	6491/34 840	Reference	Reference
Pharmacy cohort	6699/34 840	1.04	1.00–1.08
Subgroup 1 (influenza on index date)^a^			
Ages 4 to 8 years			
Primary care cohort	1113/5601	Reference	Reference
Pharmacy cohort	1080/5601	0.94	0.86–1.03
Ages 9 to 17 years			
Primary care cohort	6254/33 421	Reference	Reference
Pharmacy cohort	6461/33 421	1.04	1.00–1.08
Subgroup 2 (non-influenza vaccine[s] only on index date)^b^			
Ages 9 to 17 years^c^			
Primary care cohort	237/1419	Reference	Reference
Pharmacy cohort	238/1419	1.01	0.83–1.22
Secondary analysis^d^			
Ages 4 to 8 years			
Primary care cohort	3919/5616	Reference	Reference
Pharmacy cohort	4100/5616	1.18	1.08–1.28
Ages 9 to 17 years			
Primary care cohort	22 166/34 840	Reference	Reference
Pharmacy cohort	23 345/34 840	1.17	1.13–1.21
Subgroup 3 (influenza on index date)			
Ages 4 to 8 years			
Primary care cohort	3926/5624	Reference	Reference
Pharmacy cohort	4110/5624	1.18	1.09–1.29
Ages 9 to 17 years			
Primary care cohort	21 497/33 421	Reference	Reference
Pharmacy cohort	22 714/33 421	1.184	1.146–1.223
Subgroup 4 (non-influenza vaccine[s] only on index date)			
Ages 9 to 17 years^c^			
Primary care cohort	669/1419	Reference	Reference
Pharmacy cohort	631/1419	0.90	0.77–1.04

^a^Individuals without an influenza claim on the index date were removed from the analytical sample.

^b^Individuals with an influenza claim on the index date were removed from the analytical sample.

^c^Analysis not conducted in the younger age group due to insufficient sample size.

^d^Timely well-child visit was defined as having a well-child visit within 15 months after the qualifying well-child visit. Secondary analysis was conducted among a subset of individuals from the primary analysis who had 15 months of continuous enrollment after the index date.

### Secondary analysis: timely well-child visit within 15 months of qualifying well-child visit

For both age groups, children who received a routine vaccination at a pharmacy were more likely to have a well-child visit claim within 15 months compared with children who received a routine vaccination in a primary care setting (ages 4–8 years: OR = 1.18; 95% CI: 1.09–1.28; ages 9–17 years: OR = 1.17; 95% CI: 1.13–1.21). The association remained statistically significant among those who received an influenza vaccine on their index date (subgroup 3) across both age groups (ages 4–8 years: OR = 1.18; 95% CI: 1.09–1.29; ages 9–17 years: OR = 1.18; 95% CI: 1.15–1.22). In individuals who did not receive an influenza vaccine on their index date (subgroup 4), the likelihood of a timely well-child visit was similar in the pharmacy and primary care cohorts (OR = 0.90; 95% CI: 0.77–1.04).

## Discussion

To our knowledge, this is the first national study using claims data to evaluate the association between pharmacy-based pediatric vaccination and the likelihood of receiving a timely well-child visit. Our analyses of more than 80 000 children did not show any differences in timeliness of well-child visits based on whether they received vaccinations in pharmacies or primary care settings. We did not find any differences in association by age group or influenza vs non-influenza vaccines. As well-child visits are recommended annually, many patients may schedule their annual visit 1 to 2 months before or after 12 months from their previous visit. A slight change in schedule is unlikely to have any clinical significance and may be desired by some patients for practical purposes or to align their visit with ACIP immunization schedules. When we accounted for this by expanding the definition of timely well-child visits as those that occurred within 15 months of the qualifying visit, we saw that children who received a pharmacy-based vaccination were more likely to have a timely well-child visit compared with those who received their vaccines in a primary care setting.

Our findings suggest that parents seeking vaccination for their children at pharmacies may have health care–seeking behaviors that make them equally or more likely to receive a timely well-child visit compared with those who do not seek such pharmacy-based services. A recent study using claims data found a pediatrician visit within the past 6 months to be predictive of receiving vaccination at a pharmacy.^9^ Parents who obtain health services for their children at pharmacies might not view pharmacy-based services as substituting care, but rather reflect a selection of parents who are more proactive in the health care of their children. Survey studies have found that parents do not view pharmacies as a substitution for preventive care visits with primary care providers.^25,26^ Other perceptual factors may also affect parental decisions to seek vaccination at pharmacies. A cross-sectional online survey found that parents’ positive perception of pharmacies’ service quality and belief in vaccine efficacy play a role in their willingness to have their children vaccinated at a pharmacy.^27^

Pharmacies are more accessible than primary care settings for patients in the United States, which has resulted in greater vaccination rates among adults.^28-30^ A 2018 study found that parents perceive pharmacies to be more accessible for childhood vaccinations than pediatrician offices.^26^ A recent survey also found that younger generations of parents were more likely to seek routine vaccinations for their children in pharmacies compared with older adults.^31^ This generational attitudinal shift is likely due to a broader pattern of community pharmacies playing a greater role in patient care.^32^ Expanding routine pediatric vaccinations to pharmacies can help redistribute workload, which often leads to burnout among primary care providers.^33^

As up-to-date pediatric vaccination rates have lagged after the COVID-19 pandemic,^34^ the debate over whether pharmacists’ immunization authority should include the pediatric population has resurfaced. Several recent bills introduced in state legislatures to expand pharmacy-based vaccinations to younger age groups have faced opposition from state medical associations.^35-37^ Although a step in the right direction, relaxing age restrictions on pharmacy-based vaccinations at a statewide level is insufficient. Without greater pharmacy participation in the VFC program, this step may also worsen health disparities.

Poorer access to primary care is disproportionately concentrated among communities with lower socioeconomic status and fewer resources.^38-40^ These children are more likely to be covered under Medicaid and CHIP, and therefore likely receive their routine vaccinations under the VFC program. The VFC program serves to increase access to vaccines for children who are uninsured or underinsured, covered by Medicaid, or are part of Indigenous communities. Vaccines purchased through VFC account for approximately 50% of the doses administered to children throughout the country.^41^

Until recently, the regulatory language used by the Centers for Disease Control and Prevention (CDC) restricts VFC enrollment to providers who are authorized by the state to prescribe and administer the vaccines, an authority only conferred to pharmacists by 11 states.^42^ Further confounding the issue is the lack of clarity provided by states on how pharmacies can register in the VFC program. Despite the Department of Health and Human Services’ (HHS's) expansion of pharmacists’ authority to immunize children during the COVID-19 pandemic, the CDC had clarified that VFC program enrollment requirements remained unchanged, and continued to rely on state-specific definition of “providers.”^42^ This status quo largely prevented pharmacies from providing vaccinations to one of the most socially vulnerable groups in the country. Implications of such restrictions were especially dire during the height of the pandemic, when the well-child visit attendance rates reached record low levels for Black and Native populations.^43^

Pharmacies can play a greater role in providing necessary vaccines to patients who may otherwise not receive them. Increasing access to vaccines for all children equitably will require allowing easier pharmacy participation in the VFC program. As the COVID-19 PHE has wound down, the flexibility afforded to parents during the pandemic was recently expanded by HHS through 2029 for influenza and COVID-19 vaccines, but challenges remain in recovering vaccination rates for all routine vaccines to pre-pandemic levels. Advancing pediatric public health and increasing routine vaccinations would require a 2-step policy solution that includes expansion of pharmacies’ vaccination authority to broader age groups and participation in the VFC program by state policymakers. Future studies should explore heterogeneity in the impact of pharmacy-based vaccination across vulnerable communities and among those who receive vaccination through the VFC program.

### Strengths and limitations

Our study had many strengths, including a large, geographically diverse sample size that consisted of both pharmacy and outpatient primary care claims. The large sample size allowed the use of exact matching on the covariates. We conducted a subgroup analysis by influenza vaccine use to explore the association between receipt of a seasonal vaccine like influenza with a timely well-child visit. Finally, by modifying the definition of a timely well-child visit to 15 months from the preceding visit, we were able to assess our research question in more pragmatic scenarios where a small delay in scheduling a well-child visit may be both logistically convenient and clinically acceptable.

This study also has several limitations to consider. By requiring continuous enrollment for 12 months prior to their index date and 15 months after the index date, we have limited our sample to individuals whose parents do not experience a lapse in employer-sponsored health insurance. To explore whether patients are using pharmacy-based vaccinations to substitute existing primary care, we limited our sample to those with health insurance and evidence of receipt of well-child visits. Thus, generalizability to those without commercial health insurance, poor access to primary care services, or otherwise low participation in primary care services is limited. An additional limitation with requiring receipt of a well-child visit within 12 months preceding the index date is the selection of healthier individuals within both cohorts. We also could not explore the association between setting of vaccine receipt and timely well-child visit under a different length of the timely well-child visit definition, as times exceeding 15 months would have coincided with the COVID-19 PHE for some patients. Additionally, as Marketscan does not include race, ethnicity, or zip-code–level location data, we were unable to explore differential impact of vaccination setting on timely well-child visits among groups that have been identified as most at risk for delayed or missed immunizations and primary care visits.^39-41^ Finally, the claims data in our analyses may be reflecting varying degrees of parental health behaviors as parents are health care decision-makers for their children's vaccinations. In the older age group, we could not explore the impact of minor consent laws for vaccination by states. In some states, minor consent laws allow minors above a certain age to receive vaccines without parental consent.^44^

## Conclusion

Our study showed similar rates of timely well-child visits among children who receive routine vaccines at pharmacies and primary care settings. Our findings suggest that individuals who received a pediatric vaccine at a community pharmacy were not more likely to have a missed or delayed well-child visit.

## Supplementary Material

qxaf028_Supplementary_Data
