# Supplementary material for: Pediatric vaccination in pharmacies is not associated with delayed well-child visits among commercially insured children
Source: Health Aff Sch. 2025 Feb 10;3(2):qxaf028. doi: 10.1093/haschl/qxaf028 (PMC11837177; doi:10.1093/haschl/qxaf028)
Supplement: qxaf028_Supplementary_Data [file qxaf028_supplementary_data.zip › Supplement - 2.0.docx]

Supplemental Materials

Table of Contents

[Section 1 3](#_Toc187069611)

[Regression Specification 3](#_Toc187069612)

[Strengthening the Reporting of Observational Studies in Epidemiology (STROBE) reporting guidelines checklist 4](#_Toc187069613)

[Table A1. Well-child visit claim identification 5](#_Toc187069614)

[Table A2. Target vaccine identification in outpatient claims 6](#_Toc187069615)

[Table A3. Vaccine identification in pharmacy claims 7](#_Toc187069616)

[Table A4. Outpatient Settings Included and Excluded from Analytical Sample 8](#_Toc187069617)

[Table A5. Inclusion Criteria 9](#_Toc187069618)

[Table A6. Number of patients with index dates in 2017 and 2018 among pharmacy and primary care cohorts prior to matching 10](#_Toc187069619)

[Table A7. Primary and subgroup Analyses 11](#_Toc187069620)

[Section 2 12](#_Toc187069621)

[Covariate Selection 12](#_Toc187069622)

[Table A8 – Distribution of Index Vaccines by Age Groups and Settings 14](#_Toc187069623)

[Table A9. Patient Distribution Across States in the Primary Analysis Group. 15](#_Toc187069624)

# Section 1

## Regression Specification

$$\mathrm{logit}\left( p \right)=\alpha_{1}M_{1}+\alpha_{2}M_{2}+\ldots+a_{K}M_{K}+\beta_{1}\mathrm{Cohort}$$

Where there are *K* exactly matched pairs, each $M_{j}$ is an indicator of belonging to pair *j*, and Cohort is an indicator for pharmacy and primary care cohorts. Primary care cohort was coded as “0” and pharmacy cohort was coded as “1”. Only the $\beta_{1}$ coefficient is estimated in the conditional logistic regression model fitting procedure.

In the primary analysis, a conditional logistic regression was used where the binary outcome of *“having a timely well-child visit”* was “1” when a claim for a well-child visit was observed within twelve months of the qualifying well-child visit, and “0” if such a claim were not observed.

In the secondary analysis, a conditional logistic regression was used where the binary outcome of “*having a timely well-child visit”* was “1” when a claim for a well-child visit was observed within fifteen months of the qualifying well-child visit, and “0” if such a claim were not observed.

The covariates of interest (sex, rurality, age strata, pediatric comorbidity index, and influenza index vaccine, year of index date) were used to match the primary care and inpatient cohorts exactly.

## Strengthening the Reporting of Observational Studies in Epidemiology (STROBE) reporting guidelines checklist

|  | Item No | Recommendation | Page No |
| --- | --- | --- | --- |
| **Title and abstract** | 1 | (*a*) Indicate the study’s design with a commonly used term in the title or the abstract | 1 |
|  |  | (*b*) Provide in the abstract an informative and balanced summary of what was done and what was found |  |
| Introduction | | | |
| Background/rationale | 2 | Explain the scientific background and rationale for the investigation being reported | 4,5 |
| Objectives | 3 | State specific objectives, including any prespecified hypotheses | 6 |
| Methods | | | |
| Study design | 4 | Present key elements of study design early in the paper |  |
| Setting | 5 | Describe the setting, locations, and relevant dates, including periods of recruitment, exposure, follow-up, and data collection |  |
| Participants | 6 | (*a*) Give the eligibility criteria, and the sources and methods of selection of participants. Describe methods of follow-up | 7,8,9 |
|  |  | (*b*) For matched studies, give matching criteria and number of exposed and unexposed |  |
| Variables | 7 | Clearly define all outcomes, exposures, predictors, potential confounders, and effect modifiers. Give diagnostic criteria, if applicable | 10,11,12 |
| Data sources/ measurement | 8* | For each variable of interest, give sources of data and details of methods of assessment (measurement). Describe comparability of assessment methods if there is more than one group | 6,7 |
| Bias | 9 | Describe any efforts to address potential sources of bias | 12,13 |
| Study size | 10 | Explain how the study size was arrived at | Figure 2 |
| Quantitative variables | 11 | Explain how quantitative variables were handled in the analyses. If applicable, describe which groupings were chosen and why | 10,11 |
| Statistical methods | 12 | (*a*) Describe all statistical methods, including those used to control for confounding | 11,12,13 |
|  |  | (*b*) Describe any methods used to examine subgroups and interactions |  |
|  |  | (*c*) Explain how missing data were addressed |  |
|  |  | (*d*) If applicable, explain how loss to follow-up was addressed |  |
|  |  | (*e*) Describe any sensitivity analyses |  |
| Results | | |  |
| Participants | 13* | (a) Report numbers of individuals at each stage of study—eg numbers potentially eligible, examined for eligibility, confirmed eligible, included in the study, completing follow-up, and analysed | Figure 2 |
|  |  | (b) Give reasons for non-participation at each stage |  |
|  |  | (c) Consider use of a flow diagram |  |
| Descriptive data | 14* | (a) Give characteristics of study participants (e.g. demographic, clinical, social) and information on exposures and potential confounders | Table 1 |
|  |  | (b) Indicate number of participants with missing data for each variable of interest |  |
|  |  | (c) Summarise follow-up time (eg, average and total amount) |  |
| Outcome data | 15* | Report numbers of outcome events or summary measures over time | Table 2 |

## Table A1. Well-child visit claim identification

The CPT codes for well-child visits were identified from “Coding for Preventive Care” guidance documents by the American Academy of Pediatrics from years 2016-2019.

| **Description** | **CPT codes** |
| --- | --- |
| New patient, 1-4 years | 99382 |
| New patient, 5-11 years | 99383 |
| New patient, 12-17 years | 99384 |
| New patient, 18 or above | 99385 |
| Established patient, 1-4 years | 99392 |
| Established patient, 5-11 years | 99393 |
| Established patient, 12 to 17 years | 99394 |
| Established patient, 18 or above | 99395 |

## Table A2. Target vaccine identification in outpatient claims

The CPT codes for target vaccines were identified from “Coding for Preventive Care” guidance documents by the American Academy of Pediatrics from years 2016-2019. To exclude individuals who received immunization at the pharmacy, following additional vaccines were identified in pharmacy claims for exclusion from primary care cohort – haemophilus influenzae type B (Hib), pneumococcal (PCV13), inactivated poliovirus, hepatitis A, hepatitis B and rotavirus.

| **Description** | **CPT codes** |
| --- | --- |
| MMR | 90707 (M-M-R ii)  90710 (Proquad MMRV) |
| Varicella | 90716 (varivax)  90710 (Proquad MMRV) |
| DTaP | 90696 (Kinrix for 4-6yo)  90697  90698 (pentacel)  90700 (daptacel,infanrix for under 7yo)  90702 (DT for under 7yo)  90723 (Mediatrix) |
| Influenza (includes 2016-2023) | 90672 (Flumist quad)  90674 (Flucelvax quad)  90658 (Afluria, Flulaval, Fluzone, Fluvirin),  90672 (Flumist quadrivalent),  90630 (Fluzone quad intradermal adults),  90655 (fluzone no preservative ped),  90656 (Afluria, Fluzone No pres, Fluvirin, Fluarix, Flulaval),  90657 (Fluzone),  90685 (Fluzone quadrivalent under 3 yo),  90686 (Fluzone and Fluarix Quadrivalent),  90687 (Fluzone quadrivalent under 3yo)  90688 (fluzone and fluarix quadrivalent with preserv)  90673 (flublok vial)  90682 (flublok syringe)  90749 (Flucelvax syringe)  90756 (Flucelvax vial) |
| Meningococcal ^a^ | 90644 (MenHibrix)  90619 (Menquad)  90620 (Bexsero)  90621 (Trumenba)  90733 (Menomune)  90734 (Menactra/Menveo) |
| HPV ^b^*.* | 90649 (Gardasil)  90650 (Cervarix)  90651 (Gardasil-9) |
| Tdap | 90714 (Tenvac for 7 and up)  90715 (Adacel and Boostrix) |
| IPV | 90713 (Inactivated poliovirus vaccine) |

a. Included Menhibrix as its last shipment was in 2016, potentially used in 2017.

b. Included Gardasil-9 and Cervarix as they had their last shipments in 2016, potentially used in 2017.

## Table A3. Vaccine identification in pharmacy claims

All NDCs were identified from the U.S. Food and Drug Administration’s National Drug Code Structured Product List Data Elements (NSDE) file.^[[1]](#footnote-1)^[^1^](https://sciwheel.com/work/citation?ids=15588218&pre=&suf=&sa=0&dbf=0) Analysis focused on identification of the NDC-9, excluding last two digits of the NDC from consideration.

| **Description** | **National Drug Codes (NDC)** |
| --- | --- |
| Influenza | 49281-515-00,70461-301-10,49281-414-10,19515-896-01,19515-901-41,19515-908-41,49281-621-78,66019-306-01,19515-912-52,49281-712-48,49281-719-10,42874-015-10,49281-181-00,49281-514-25,66019-302-01,58160-896-41,42874-117-10,49281-515-25,19515-897-01,49281-417-10,70461-119-12,42874-117-01,49281-625-78,49281-416-88,19515-903-11,33332-319-01,70461-319-03,66521-118-11,19515-903-01,19515-850-52,49281-710-48,49281-415-88,49281-419-10,19515-908-52,33332-317-02,49281-405-88,19515-898-11,42874-016-10,49281-710-40,49281-401-65,49281-417-50,49281-629-78,33332-319-02,49281-399-65,58160-883-52,49281-414-50,58160-883-41,49281-181-25,58160-898-52,43835-0020-2,49281-320-88,49281-519-25,43835-0020-1,49281-336-15,49281-631-15,33332-417-11,33332-219-21,58160-896-52,42874-015-01,49281-623-78,19515-897-11,70461-200-11,49281-417-88,58160-898-41,70461-201-01,49281-331-15,49281-397-65,49281-516-00,33332-317-01,49281-415-50,49281-418-58,58160-907-41,33332-318-01,49281-419-88,49281-712-40,49281-517-25,19515-900-11,66019-305-01,49281-116-00,49281-519-00,49281-331-78,49281-417-58,49281-627-78,42874-017-01,49281-414-58,49281-621-15,19515-906-41,33332-418-10,58160-905-41,49281-116-25,70461-418-11,19515-850-41,76420-495-01,49281-316-50,70461-201-11,49281-397-88,58160-903-52,70461-119-02,49281-396-15,49281-516-25,49281-718-88,49281-629-15,66019-304-10,49281-419-58,49281-518-25,49281-399-88,49281-631-78,33332-419-10,49281-719-88,70461-200-01,49281-396-78,76420-517-01,66019-303-01,19515-912-41,33332-419-11,49281-401-88,49281-627-15,19515-909-52,70461-319-04,19515-845-01,19515-900-01,66019-304-01,49281-517-00,33332-219-20,19515-845-11,49281-418-50,33332-417-10,19515-898-01,49281-403-88,66521-118-02,49281-405-65,66019-305-10,49281-518-00,70461-119-11,49281-415-10,49281-403-65,66019-303-10,49281-336-78,49281-416-50,19515-906-52,49281-418-88,42874-016-01,66521-118-10,66521-118-12,49281-625-15,42874-017-10,70461-318-04,58160-903-41,19515-896-11,49281-416-58,49281-415-58,49281-418-10,33332-318-02,19515-909-41,70461-318-03,66019-306-10,66019-302-10,49281-316-88,49281-320-50,70461-419-10,19515-901-52,70461-419-11,49281-514-00,70461-301-12,58160-905-52,58160-907-52,49281-708-48,70461-418-10,49281-418-00,49281-623-15,49281-416-10,49281-718-10,33332-418-11,49281-419-50,70461-119-10,49281-708-40,49281-414-88 |
| DTaP | 49281-225-58,58160-811-51,58160-810-51,58160-810-41,49281-286-01,49281-511-05,49281-225-10,58160-810-46,49281-286-10,49281-562-10,49281-564-15,58160-811-41,58160-812-41,49281-564-88,49281-510-05,58160-810-01,58160-810-11,58160-812-01,49281-564-10,58160-812-46,58160-811-11,49281-564-58,58160-811-46,49281-286-05,58160-810-43,49281-562-58,58160-812-11,58160-812-51,50090-2883-9,49281-278-10,58160-811-01,50090-2883-0,58160-811-43,58160-810-52,49281-286-58,58160-812-52,58160-812-43,58160-811-52 |
| MMR | 0006-4826-01,0006-4999-01,0006-4827-01,54868-0980-0,50090-1860-0,0006-4171-00,0006-4681-00,0006-4171-01,50090-1860-9,0006-4826-00,0006-4999-00,58160-824-15,0006-4827-00,0006-4681-01 |
| Varicella | 0006-4826-01,0006-4999-01,0006-4827-01,0006-4171-00,0006-4171-01,0006-4826-00,0006-4999-00,0006-4827-00 |
| Tdap | 50090-1831-0,49281-400-20,49281-400-05,58160-842-11,50090-1377-9,49281-400-10,58160-842-43,0006-4133-01,49281-400-89,17478-131-01,17478-131-00,58160-842-46,21695-413-01,58160-842-32,50090-1377-0,49281-400-58,58160-842-01,49281-400-88,50090-1377-2,58160-842-51,52125-848-08,50090-1377-1,58160-842-52,50090-1831-9,61786-433-57,58160-842-05,49281-400-15,58160-842-34,0006-4133-41,58160-842-41,52125-848-57 |
| Meningococcal ^a^ | 0005-0100-01,46028-208-02,49281-489-91,49281-590-05,0005-0100-05,58160-827-03,0005-0100-10,49281-589-58,49281-590-58,58160-809-01,58160-976-20,58160-801-11,50090-6180-0,49281-489-10,58160-976-02,49281-489-04,0005-0100-02,49281-589-05,50090-6180-1,58160-955-09,49281-489-01,58160-827-30,46028-114-01,46028-114-02,46028-208-01,52125-836-57,46028-114-11,58160-809-05,58160-976-06 |
| HPV ^b^ | 50090-4958-0,58160-830-52,52125-833-01,58160-830-43,0006-4121-02,0006-4109-02,0006-4045-00,0006-4119-03,58160-830-11,58160-830-32,50090-1523-9,0006-4109-09,0006-4121-01,0006-4119-01,50090-1523-1,0006-4109-01,58160-830-41,58160-830-05,58160-830-34,50090-2443-0,0006-4045-41,0006-4045-01,58160-830-46,0006-4119-02,58160-830-01,0006-4109-06,50090-4958-1 |
| IPV | 49281-0860-10, 49281-0860-78, 49281-0860-55, 49281-0860-88, 50090-1693-0, 50090-1693-9 |

a. Included Menhibrix as its last shipment was in 2016, potentially used in 2017.

b. Included Gardasil-9 and Cervarix as they had their last shipments in 2016, potentially used in 2017.

## Table A4. Outpatient Settings Included and Excluded from Analytical Sample

| **Included Outpatient Settings** | **Excluded outpatient settings** |
| --- | --- |
| Office | Emergency Room – Hospital |
| Outpatient Hospital on Campus | Walk in Retail Health Clinic |
| State/Local Public Health Clinic | Other/Unknown |
| Rural Health Clinic | Urgent Care Facility |
| Federally Qualified Health Center | Mobile Unit |
| Independent Clinic | School |
| Outpatient | Mass Immunization Center |
| Outpatient Hospital Off Campus | Residential Substance Abuse Facility |
|  | Inpatient Hospital |
|  | Pharmacy |
|  | Patient home |
|  | Independent Laboratory |
|  | Military Treatment Facility |
|  | Ambulatory Surgical Center |
|  | Birthing Center |
|  | Community Mental Health Center |
|  | Psychiatric Residential Treatment Center |
|  | Comprehensive outpatient rehab facility |
|  | Nursing Facility |
|  | Psych Facility Partial Hospital |
|  | End Stage Renal Disease Facility |

## Table A5. Inclusion Criteria

| Criteria | Pharmacy Cohort | Primary Care Cohort | Rationale |
| --- | --- | --- | --- |
| Must have a claim for a target vaccine | X | X | NDCs in pharmacy claims and CPT codes in outpatient claims data were identified for target vaccines |
| Must have continuous enrollment before and after the index date | X | X | All patients must have had 12 months of continuous enrollment before and after their index date. Continuous enrollment will ensure that all patients had opportunity to have claims for well-child visits before and after their index dates. For modified outcome analyses (secondary analyses, including subgroup analyses 3 and 4), individuals with 15 months of continuous enrollment after their index date were included to ensure a well-child visit within 15 months after index date could be observed. To avoid inducing healthy patient bias, we applied the same criterion to the primary analysis cohort. |
| Must have a claim for a well-child visit within 12 months preceding index-date | X | X | To ascertain whether a well-child visit after index date was timely, identification of a well-child visit prior to index date was necessary. Without this anchor well-child visit, we would not be able to discern a timely well-child visit from a delayed one. Additionally, opposers of pharmacy-based pediatric vaccinations suggest that permitting younger children to receive routine vaccinations at pharmacies will result in missed well-child visits. Children who do not have access to a primary care provider would not be at risk of missing a well-child visit, and this requirement establishes that the patient has access to primary care. |
| Must not have an inpatient visit within 3 months preceding the index date | X | X | Individuals with recent inpatient admissions may be more likely to follow up regularly with a physician in outpatient setting and incidentally receive routine vaccination during the visit. |
| Exclusion of individuals whose index vaccine was received in outpatient settings where primary care could not be provided | N/A | X | We excluded individuals whose index vaccines were in settings where they could not receive preventive care (e.g., retail clinics, mobile vaccination units, school). |
| Exclusion of individuals with vaccines in pharmacy claims within 12 months preceding index date |  | X | This criterion avoided inclusion of individuals in both cohorts. We did not exclude those with outpatient vaccination claims in from the pharmacy cohort to improve our external validity. |

## Table A6. Number of patients with index dates in 2017 and 2018 among pharmacy and primary care cohorts prior to matching

| Year of the index date | Primary Care  (4 to 8 years old) | Pharmacy  (4 to 8 years old) | Primary Care  (9 to 17 years old) | Pharmacy  (9 to 17 years old) |
| --- | --- | --- | --- | --- |
| 2017 | 307,096 (64.59%) | 3,055 (34.01%) | 579,733 (68.60%) | 21,489 (39.24%) |
| 2018 | 168,329 (35.41%) | 5,927 (65.99%) | 265,345 (31.40%) | 33,280 (60.76%) |

## Table A7. Primary and subgroup Analyses

| **Group** | **Vaccines** | **Age group(s)** | **Definition of timely well-child visit** |
| --- | --- | --- | --- |
| Primary analysis | All target vaccines | 4-8 years; 9-17 years | Follow-up well-child visit within 12 months of qualifying well-child visit ^c^ |
| Subgroup 1 | Influenza on index date ^a^ |  |  |
| Subgroup 2 | No influenza on index date | 9-17 years ^b^ |  |
| Secondary analysis | All target vaccines | 4-8 years; 9-17 years | Follow-up well-child visit within 15 months of qualifying well-child visit ^c^ |
| Subgroup 3 | Influenza on index date ^a^ |  |  |
| Subgroup 4 | No influenza on index date | 9-17 years ^b^ |  |

a. Concomitant receipt of non-influenza vaccine on index date did not result in exclusion from analysis.
b. Younger age group (4 to 8 years) had low sample size for inferential statistics in subgroup analyses 2 and 4.
c. The qualifying well-child visit was the anchor well-child visit used as a selection criterion for the study. The follow-up well-child visit was used to ascertain timeliness of the well-child visit.

# Section 2

## Covariate Selection

We matched primary care and pharmacy cohorts on several covariates that were identified a-priori. A detailed description of these covariates is included below.

**Rurality** – Access and availability of health services can vary by rurality, where some patients in rural areas may rely more on pharmacies for immunizations compared to individuals in urban areas.^[[2]](#footnote-2),^^[[3]](#footnote-3)^ Marketscan consists of Metropolitan Statistical Areas (MSA) within patient demographics. Individuals without a MSA value presumably reside in rural areas, and this approach has been adopted in other studies.^[[4]](#footnote-4),^^[[5]](#footnote-5),^^[[6]](#footnote-6)^

**Pediatric Comorbidity Index** – Individuals with a higher comorbidity burden may be more likely to have follow up visits with their providers and may incidentally receive essential preventive services like immunizations during these visits. The pediatric comorbidity index was developed using Marketscan data based on risk of hospitalization in patients.[^6^](https://sciwheel.com/work/citation?ids=14445168&pre=&suf=&sa=0&dbf=0)

**State –** States have disparate laws and regulations on patient ages, as well as the types of vaccines that can be administered at a pharmacy within a community setting. We matched the cohorts on states exactly to control for any state-level effects.

**Influenza vaccine indicator –** As influenza vaccine is seasonal, it may affect patient behavior in seeking vaccination at the pharmacy if the annual well-child visit does not align with the influenza season. To control for this season effect, we matched the two cohorts on whether influenza was among the index-vaccines (i.e., a target vaccine received on index date). Those with the influenza indicator of “1” were those who had influenza as at least one of the vaccines on their index date. Those with the indicator of “0” were those who did not have influenza among their index vaccines.

**Age subgroups -** Within the younger (ages 4 to 8 years) and the older (ages 9 to 17 years) age groups, we further identified subgroups based on the ACIP immunizations schedule. In the younger age group, we matched the primary care and pharmacy cohorts on ages 4 to 6 years and 7 to 8 years. In the older age group, we matched the primary care and pharmacy cohorts on ages 9 to 13 years and 14 to 17 years.

**Index year –** We descriptively examined the distribution of index dates in both cohorts between the study years and observed greater concentration of index dates in 2018 in the pharmacy cohort (Table A6). We also observed disproportionately fewer index dates in 2018 for the older primary care cohort. To control for potential time related effects, we controlled for the year of the index vaccine.

**Health Plan Type –** There may be differences in primary care provider networks, cost-sharing and pharmacy access based on different type of health plans. To account for some of the access related issues that may drive patient health care seeking behavior, we matched the pharmacy and primary care cohorts on health plan type.

**Sex** – We controlled for sex to account for differences in health care seeking behavior that may be driven by patients’ sex. This may include perceived importance of certain routine vaccines like the HPV vaccine for biologically female patients vs. male patients.

## Table A8. Distribution of Index Vaccines by Age Groups and Settings

|  | **Ages 4 to 8 years** | | **Ages 9 to 17 years** | |
| --- | --- | --- | --- | --- |
| **Index Vaccine** | *Primary Care Cohort (n=5,611)* | *Pharmacy Cohort (n=5,611)* | *Primary Care Cohort (n=34,836)* | *Pharmacy Cohort (n=34,836)* |
| Influenza | 5,599 (99.79%) | 5,599 (99.79%) | 33,597 (96.44%) | 33,484 (96.12%) |
| Varicella | 303 (5.40%) | 0 (0%) |  |  |
| MMR | 292 (5.20%) | 14 (0.25%) |  |  |
| MMR and Varicella^a^ | 546 (9.73%) | 1 (0.02%) |  |  |
| DTAP | 845 (15.06%) | 9 (0.16%) |  |  |
| IPV | 149 (2.66%) | 0 (0%) |  |  |
| Tdap |  |  | 1,861 (5.34%) | 356 (1.02%) |
| Meningococcal |  |  | 4,567 (13.11%) | 782 (2.24%) |
| HPV |  |  | 842 (2.42%) | 477 (1.37%) |

^a^ Claims for combination MMRV vaccine.
Grey section indicates that respective vaccine not included among target vaccines for the age range.
Sum of percentages in each column may exceed 100% as several index vaccines for same individuals are listed.
MMR = Measles, Mumps and Rubella; DTAP = Diphtheria, Tetanus and Acellular Pertussis; IPV = Inactivated Poliovirus; Tdap = Tetanus, diphtheria and acellular pertussis; HPV = Human Papillomavirus

## Table A9. Patient Distribution Across States in the Primary Analysis Group.

| **State** | **Ages 4 to 8 years** | | **Ages 9 to 17 years** | |
| --- | --- | --- | --- | --- |
|  | Pharmacy Cohort (n=5,616) | Primary Care Cohort (n=5,616) | Pharmacy Cohort (n=34,836) | Primary Care Cohort (n=34,836) |
| Alabama | 157 (2.80%) | | 560 (1.61%) | |
| Alaska | 0 (0%) | | 3 (0.01%) | |
| Arizona | 168 (2.99%) | | 705 (2.02%) | |
| Arkansas | 7 (0.12%) | | 77 (0.22%) | |
| California | 425 (7.57%) | | 2050 (5.88%) | |
| Colorado | 228 (4.06%) | | 875 (2.51%) | |
| Connecticut | 1 (<0.1%) | | 65 (0.19%) | |
| Delaware | 62 (1.1%) | | 470 (1.35%) | |
| Florida | 5 (<0.1%) | | 88 (0.25%) | |
| Georgia | 220 (3.92%) | | 1727 (4.96%) | |
| Idaho | 26 (0.46%) | | 78 (0.22%) | |
| Illinois | 5 (<0.1%) | | 1112 (3.19%) | |
| Indiana | 22 (0.39%) | | 715 (2.05%) | |
| Iowa | 116 (2.07%) | | 422 (1.21%) | |
| Kansas | 100 (1.78%) | | 428 (1.23%) | |
| Kentucky | 66 (1.18%) | | 1155 (3.32%) | |
| Louisiana | 42 (0.75%) | | 178 (0.51%) | |
| Maine | 8 (0.14%) | | 37 (0.11%) | |
| Maryland | 6 (0.11%) | | 407 (1.17%) | |
| Massachusetts | 11 (0.2%) | | 868 (2.49%) | |
| Michigan | 524 (9.33%) | | 2154 (6.18%) | |
| Minnesota | 73 (1.3%) | | 335 (0.96%) | |
| Mississippi | 19 (0.34%) | | 102 (0.29%) | |
| Missouri | 62 (1.1%) | | 643 (1.85%) | |
| Montana | 0 (0%) | | 9 (<0.1%) | |
| Nebraska | 54 (0.96%) | | 142 (0.41%) | |
| Nevada | 53 (0.94%) | | 159 (0.46%) | |
| New Hampshire | 11 (0.2%) | | 79 (0.23%) | |
| New Jersey | 27 (0.48%) | | 577 (1.66%) | |
| New Mexico | 20 (0.36%) | | 67 (0.19%) | |
| New York | 393 (7%) | | 1260 (3.62%) | |
| North Carolina | 7 (0.12%) | | 947 (2.72%) | |
| North Dakota | 3 (<0.1%) | | 1 (0%) | |
| Ohio | 296 (5.27%) | | 2294 (6.59%) | |
| Oklahoma | 36 (0.64%) | | 183 (0.53%) | |
| Oregon | 122 (2.17%) | | 852 (2.45%) | |
| Pennsylvania | 10 (0.18%) | | 889 (2.55%) | |
| Rhode Island | 3 (<0.1%) | | 10 (<0.1%) | |
| South Carolina | 30 (0.53%) | | 459 (1.32%) | |
| South Dakota | 20 (0.36%) | | 60 (0.17%) | |
| Tennessee | 122 (2.17%) | | 483 (1.39%) | |
| Texas | 588 (10.47%) | | 3995 (11.47%) | |
| Utah | 71 (1.26%) | | 178 (0.51%) | |
| Virginia | 516 (9.19%) | | 1289 (3.70%) | |
| Washington | 235 (4.18%) | | 489 (1.40%) | |
| Washington, DC | 3 (<0.1 %) | | 4 (<0.1 %) | |
| West Virginia | 2 (<0.1%) | | 15 (<0.1 %) | |
| Wisconsin | 39 (0.69%) | | 235 (0.67%) | |
| Wyoming | 2 (<0.1 %) | | 3 (<0.1 %) | |
| Nation, unknown region | 600 (10.68%) | | 4903 (14.07%) | |

*Note: these states are the primary enrollee’s state of residence, and do not reflect location of pharmacies where transaction has occurred.*

1. Food and Drug Administration. NSDE (NDC SPL Data Elements File) Available from: https://www.fda.gov/industry/structured-product-labeling-resources/nsde [↑](#footnote-ref-1)
2. Saelee R, Zell E, Murthy BP, Castro-Roman P, Fast H, Meng L, et al. Disparities in COVID-19 Vaccination Coverage Between Urban and Rural Counties - United States, December 14, 2020-January 31, 2022. MMWR Morb Mortal Wkly Rep. 2022 Mar 4;71(9):335–40. [↑](#footnote-ref-2)
3. AlMahasis SO, Fox B, Ha D, Qian J, Wang C-H, Westrick SC. Pharmacy-based immunization in rural USA during the COVID-19 pandemic: A survey of community pharmacists from five southeastern states. Vaccine. 2023 Apr 6;41(15):2503–13. [↑](#footnote-ref-3)
4. Chen Z, Roy K, Khushalani JS, Puddy RW. Trend in rural-urban disparities in access to outpatient mental health services among US adults aged 18-64 with employer-sponsored insurance: 2005-2018. J Rural Health. 2022 Sep;38(4):788–94. [↑](#footnote-ref-4)
5. Graves JM, Mackelprang JL, Moore M, Abshire DA, Rivara FP, Jimenez N, et al. Rural-urban disparities in health care costs and health service utilization following pediatric mild traumatic brain injury. Health Serv Res. 2019 Apr;54(2):337–45. [↑](#footnote-ref-5)
6. Sun JW, Bourgeois FT, Haneuse S, Hernández-Díaz S, Landon JE, Bateman BT, et al. Development and validation of a pediatric comorbidity index. Am J Epidemiol. 2021 May 4;190(5):918–27. [↑](#footnote-ref-6)
